# The prognostic effects of the geriatric nutritional risk index on elderly acute kidney injury patients in intensive care units

**DOI:** 10.3389/fmed.2023.1165428

**Published:** 2023-05-11

**Authors:** Dan Liao, Yonghua Deng, Xinchun Li, Ju Huang, Jiayue Li, Ming Pu, Fenglian Zhang, Lijun Wang

**Affiliations:** ^1^Department of Nephrology, Mianyang Central Hospital, School of Medicine, University of Electronic Science and Technology of China, Mianyang, China; ^2^Department of Nephrology, Chengdu Second People's Hospital, Chengdu, China; ^3^North Sichuan Medical College, Nanchong, China; ^4^Department of Nephrology, Mianyang People's Hospital, Mianyang, China; ^5^Chengdu Medical College, Chengdu, China

**Keywords:** geriatric nutritional risk index, acute kidney injury, intensive care unit, elderly, prognosis

## Abstract

**Introduction:**

The geriatric nutritional risk index (GNRI), a nutritional screening tool specifically for the aging population, has been proven to be associated with worse outcomes in chronic kidney disease patients, especially in the hemodialysis population. However, the predictive validity of GNRI in critically ill elderly patients with acute kidney injury (AKI) is yet to be determined. This analysis sought to examine the prognostic effects of GNRI on elderly AKI patients in intensive care units (ICUs).

**Methods:**

We collected elderly AKI patient-relevant data from the Medical Information Mart for Intensive Care III database. AKI was diagnosed and staged according to the “Kidney Disease Improving Global Outcomes” criteria. In the study, 1-year mortality was considered the primary outcome, whereas in-hospital, ICU, 28-day and 90-day mortality, and prolonged length of stay in ICU and hospital were selected as the secondary outcomes.

**Results:**

In all, 3,501 elderly patients with AKI were selected for this study, with a 1-year mortality rate of 36.4%. We classified the study population into low (≤98) and high (>98) GNRI groups based on the best cutoff value. The incidence of endpoints was remarkably lower in patients with elevated GNRI (*p* < 0.001). When stratified by the AKI stage, patients with high GNRI at AKI stages 1, 2, and 3 had markedly lower 1-year mortality than those with low GNRI (all *p* < 0.05). The multivariable regression analysis identified the independent prognostic ability of GNRI on the research outcomes (all *p* < 0.05). Restricted cubic spline exhibited a linear correlation between GNRI and 1-year death (*p* for non-linearity = 0.434). The prognostic implication of GNRI on 1-year mortality was still significant in patients with the most subgroups.

**Conclusion:**

In critically ill elderly patients with AKI, elevated GNRI upon admission was strongly correlated with a lower risk of unfavorable outcomes.

## 1. Introduction

Acute kidney injury (AKI) is a frequent complication in intensive care units (ICUs) and contributes to important nutritional problems (1, 2). A previous study has reported that the caloric intake of severe AKI patients receiving renal replacement therapy is very low (3). The European Society for Parenteral and Enteral Nutrition (ESPEN) guideline strongly recommends providing nutritional support for hospitalized AKI patients (4), and some medical nutrients may facilitate the recovery of renal function in AKI patients (5). However, malnutrition remains under-recognized and underdiagnosed, particularly in older patients with frailty. It has been reported that ~ 9% of inpatients are diagnosed with malnutrition, and in fact, the incidence of malnutrition among inpatients is ~ 40% (6–8). Thus, an accurate assessment of the nutritional status is pivotal to improving malnutrition.

The geriatric nutritional risk index (GNRI), a simple and useful nutritional screening tool specifically for the aging population, has been widely used in multiple clinical scenarios, including patients with cancer, heart failure, cardio-cerebrovascular disease, and acute respiratory failure (9–14). Furthermore, a previous study revealed the prognostic implication of GNRI in critically ill patients (15). An increasing body of evidence has established the correlation between GNRI and worse outcomes in patients with chronic kidney disease (CKD) and hemodialysis (16, 17). Taken together, it is reasonable to speculate that GNRI might be correlated with the poor prognosis in AKI patients. No studies have hitherto assessed the predictive validity of GNRI in critically ill elderly patients with AKI. Accordingly, this research sought to evaluate the ability of GNRI to predict the prognosis in elderly AKI patients in the ICU.

## 2. Materials and methods

### 2.1. Data sources

This research was based on the Medical Information Mart for Intensive Care III (MIMIC-III), a freely accessible large critical-care database covering many critically ill patients at the Beth Israel Deaconess Medical Center from 2001 to 2012 (https://mimic.mit.edu/). The researcher (Yonghua Deng) received the seniority to extract data from this database after completing the required training course.

### 2.2. Population selection

Patients aged over 65 years old and diagnosed with AKI were involved in this analysis. We excluded patients who lacked serum albumin or weight or height, had repeat admissions, and stayed in the ICU for <2 days, as well as patients with pre-existing CKD.

### 2.3. Data extraction and definitions

The extracted variables contained demographic data (age, gender, weight, and height), vital signs (heart rate and blood pressure), comorbidities [such as coronary heart disease (CHD), sepsis, and liver cirrhosis], laboratory parameters at admission [such as albumin, blood urea nitrogen (BUN), and serum creatinine (SCr)], sequential organ failure assessment (SOFA), Charlson comorbidity index (CCI), treatment information [continuous renal replacement therapy (CRRT), vasopressors, and mechanical ventilation], and types of intensive care unit (ICU). The laboratory indicators were taken from the first measurement recorded after admission. BMI was defined as weight in kilograms/(height in meters)^2^. GNRI was calculated as follows: 14.89 × serum albumin (g/dl) + 41.7 × BMI/22 (18). AKI was diagnosed and staged according to the “Kidney Disease Improving Global Outcomes” criteria (19). In the study, 1-year mortality was considered the primary outcome, whereas in-hospital, ICU, 28-day and 90-day mortality, and prolonged length of stay (LOS) in the ICU and hospital were selected as the secondary outcomes. Excessive LOS was determined as the length of stay above the 75th percentile. Thus, the prolonged ICU and hospitalization LOS were more than 10 and 19 days, respectively.

### 2.4. Statistical analysis

A Kolmogorov–Smirnov test was used with continuous variables to determine whether their distributions were normal. Continuous variables were summarized as median (interquartile range) and examined by the Mann–Whitney U-test because of the skewed distribution of these variables. Categorical data were characterized as numbers with proportions, and comparisons between groups were employed using the chi-square test. We built a receiver operating characteristic (ROC) curve to determine the optimal cutoff value of GNRI for predicting 1-year death. The cutoff point was applied to divide patients into two groups. A multivariable linear regression analysis was employed to confirm the relationship between GNRI and relevant clinical variables. Furthermore, the 365-day cumulative survival between the two groups was compared using the Kaplan–Meier (KM) curve with the log-rank test. We carried out multivariable logistic and Cox proportional hazards regression analyses to verify the impact of GNRI on the adverse outcomes, adjusting for the potential confounding factors. These covariates were related to the 1-year mortality in the univariable Cox regression analyses. Meanwhile, GNRI was examined as both a continuous and a categorical variable. Restricted cubic spline (RCS) with four knots was applied to explore the curve relationship between GNRI and 365-day death. We also carried out a stratification analysis to figure out whether the predictive significance of GNRI for 1-year mortality was sustained across the different subgroups classified by gender, age, AKI stage, comorbidities, sofa score, CCI, ICU types, CRRT, vasopressors, and mechanical ventilation. We employed all statistical analyses with the R software (version 3.6.3) and MedCalc (version 19.1). A *P*-value <0.05 was regarded as statistically significant.

## 3. Results

### 3.1. Patient characteristics

In total, 3,501 elderly patients with AKI were selected for this analysis, with a 1-year mortality rate of 36.4% (Supplementary Figure 1). Patients were categorized into low (≤98) and high (>98) GNRI groups based on the optimum cutoff score determined by ROC curve analysis (the area under the curve was 0.655, and the sensitivity and specificity were 0.618 and 0.623, respectively; Supplementary Figure 2). The baseline characteristics by categories of GNRI (high and low GNRI groups) are summarized in Table 1. The two groups presented a statistically significant difference in terms of age, gender, heart rate, hypertension, diabetes, CHD, malignancy, sepsis, white blood cell count (WBC), hemoglobin, BUN, SCr, sodium, bicarbonate, aspartate aminotransferase (AST), SOFA score, CCI, ICU types, and CRRT. The low GNRI group had worse in-hospital mortality, ICU mortality, 28-day mortality, 90-day mortality, 1-year mortality, and longer ICU and hospital stay length than the high GNRI group (all *p* < 0.001, Table 1). A multivariable linear regression analysis was carried out to examine the relationship between GNRI and the relevant clinical variables. In the univariable analysis, elevated GNRI was associated with decreased age, the prevalence of malignancy and sepsis, sofa score, CCI, the use of mechanical ventilation, and increased prevalence of hypertension, diabetes, CHD, heart failure, and a higher level of hemoglobin (Table 2). The multivariable analysis confirmed that GNRI was positively related to diabetes, hypertension, CHD, heart failure, and hemoglobin and negatively related to age, sepsis, CCI, and ventilation (Table 2).

**Table 1:** Baseline characteristics of participants according to the GNRI category.

**Variables**	**Total**	**High GNRI (>98)**	**Low GNRI (≤98)**	***P***
	***N*** = **3,501**	***N*** = **1,941**	***N*** = **1,560**	
Age, years	76.7 (71.1, 82.4)	75.7 (70.3, 81.2)	78.1 (72.6, 83.6)	<0.001
Male, *n* (%)	1,880 (53.7)	1,076 (55.4)	804 (51.5)	0.022
SBP, mmHg	120 (104, 138)	119 (105, 137)	120 (103, 139)	0.937
DBP, mmHg	59.0 (50.0, 70.0)	60.0 (51.0, 70.0)	59.0 (50.0, 69.0)	0.283
Heart rate, bpm	86.0 (75.0, 98.0)	85.0 (75.0, 94.2)	88.0 (76.0, 102.0)	<0.001
Hypertension, *n* (%)	2,078 (59.4)	1,284 (66.2)	794 (50.9)	<0.001
Diabetes, *n* (%)	995 (28.4)	670 (34.5)	325 (20.8)	<0.001
CHD, *n* (%)	1,607 (45.9)	1,074 (55.3)	533 (34.2)	<0.001
Heart failure, *n* (%)	1,450 (41.4)	822 (42.3)	628 (40.3)	0.211
Liver cirrhosis, *n* (%)	123 (3.5)	70 (3.6)	53 (3.4)	0.739
Malignancy, *n* (%)	786 (22.5)	381 (19.6)	405 (26.0)	<0.001
Sepsis, *n* (%)	462 (13.2)	186 (9.6)	276 (17.7)	<0.001
WBC, k/ul	9.9 (7.2, 13.8)	9.2 (6.9, 12.9)	10.8 (7.8, 14.9)	<0.001
Hemoglobin, g/dl	11.9 (10.5, 13.2)	12.3 (10.9, 13.6)	11.4 (10.1, 12.7)	<0.001
BUN, mg/dl	22.0 (16.0, 31.0)	22.0 (16.0, 30.0)	23.0 (16.0, 33.0)	0.046
SCr, mg/dl	1.0 (0.8, 1.4)	1.0 (0.8, 1.3)	1.0 (0.8, 1.4)	0.036
Sodium, mEq/l	139 (136, 141)	139 (136, 141)	138 (135, 141)	<0.001
Potassium, mEq/l	4.1 (3.8, 4.5)	4.1 (3.8, 4.5)	4.1 (3.8, 4.6)	0.485
Anion gap, mEq/L	14.0 (13.0, 17.0)	15.0 (13.0, 17.0)	14.0 (12.0, 17.0)	0.884
Bicarbonate, mEq/L	25.0 (22.0, 27.0)	25.0 (23.0, 28.0)	24.0 (21.0, 27.0)	<0.001
ALT, u/l	23.0 (15.0, 41.0)	23.0 (16.0, 37.0)	24.0 (14.0, 45.0)	0.293
AST, u/l	31.0 (21.0, 57.0)	29.0 (20.0, 50.0)	35.0 (21.0, 66.0)	<0.001
SOFA score	5.0 (3.0, 7.0)	5.0 (3.0, 7.0)	5.0 (3.0, 8.0)	0.006
CCI	5.0 (4.0, 6.0)	5.0 (4.0, 6.0)	5.0 (4.0, 7.0)	<0.001
**ICU types**				<**0.001**
CSRU	1,271 (36.3)	869 (44.8)	402 (25.8)	
CCU	617 (17.6)	346 (17.8)	271 (17.4)	
MICU	850 (24.3)	384 (19.8)	466 (29.9)	
SICU	492 (14.1)	225 (11.6)	267 (17.1)	
TSICU	271 (7.7)	117 (6.0)	154 (9.9)	
CRRT, *n* (%)	198 (5.7)	96 (4.9)	102 (6.5)	0.043
Vasopressors, *n* (%)	2,445 (69.8)	1,376 (70.9)	1,069 (68.5)	0.130
Ventilation, *n* (%)	1,375 (39.3)	651 (33.5)	724 (46.4)	<0.001
Length of ICU stay	5.2 (3.2, 9.9)	4.7 (3.0, 8.0)	6.2 (3.6, 12.5)	<0.001
Length of hospital stay	11.9 (7.9, 18.8)	10.9 (7.8, 16.6)	13.3 (8.2, 22.0)	<0.001
In-hospital mortality	678 (19.4)	256 (13.2)	422 (27.1)	<0.001
ICU-mortality	540 (15.4)	213 (11.0)	327 (21.0)	<0.001
28-days mortality	848 (24.2)	329 (17.0)	519 (33.3)	<0.001
90-days mortality	1,005 (28.7)	391 (20.1)	614 (39.4)	<0.001
1-year mortality	1,276 (36.4)	519 (26.7)	757 (48.5)	<0.001

GNRI, geriatric nutritional risk index; SBP, systolic blood pressure; DBP, diastolic blood pressure; CHD, coronary heart disease; WBC, white blood cell count; BUN, blood urea nitrogen; SCr, serum creatinine; ALT, alanine aminotransferase; AST, aspartate aminotransferase; SOFA, sequential organ failure assessment; CCI, Charlson comorbidity index; ICU, intensive care unit; CCU, coronary care unit; CSRU, cardiothoracic surgery recovery unit; MICU, medical intensive care unit; SICU, surgical intensive care unit; TSICU, trauma and surgical intensive care unit; CRRT, continuous renal replacement therapy.

**Table 2:** Linear regression analysis for GNRI.

	**Univariable analysis**		**Multivariable analysis**	
	β **(95%CI)**	***P***	β **(95%CI)**	***P***
Age	−0.48 (−0.56, −0.41)	<0.001	−0.37 (−0.44, −0.29)	<0.001
Male	0.71 (−0.36, 1.79)	0.195		
Hypertension	5.01 (3.93, 6.09)	<0.001	3.92 (2.89, 4.94)	<0.001
Diabetes	7.01 (5.84, 8.17)	<0.001	6.07 (4.89, 7.25)	<0.001
CHD	6.51 (5.45, 7.56)	<0.001	3.55 (2.50, 4.60)	<0.001
Heart failure	1.55 (0.46, 2.64)	0.005	3.14 (2.06, 4.23)	<0.001
Liver cirrhosis	1.19 (−1.73, 4.10)	0.425		
Malignancy	−3.01 (−4.29, −1.73)	<0.001	−0.03 (−1.36, 1.29)	0.962
Sepsis	−5.98 (−7.55, −4.41)	<0.001	−3.55 (−5.08, −2.02)	<0.001
Hemoglobin	1.57 (1.32, 1.82)	<0.001	1.24 (1.00, 1.48)	<0.001
SOFA score	−0.27 (−0.44, −0.10)	0.002	−0.04 (-0.20, 0.12)	0.651
CCI	−1.01 (−1.29, −0.73)	<0.001	−0.86 (−1.19, −0.52)	<0.001
CRRT	−0.41 (−2.73, 1.91)	0.729		
Ventilation	−3.73 (−4.83, −2.64)	<0.001	−1.61 (−2.69, −0.54)	0.003
Vasopressors	0.78 (−0.39, 1.95)	0.191		

95% CI, 95% confidence interval; other abbreviations as in Table 1.

### 3.2. Clinical outcomes

**Figure 1:**
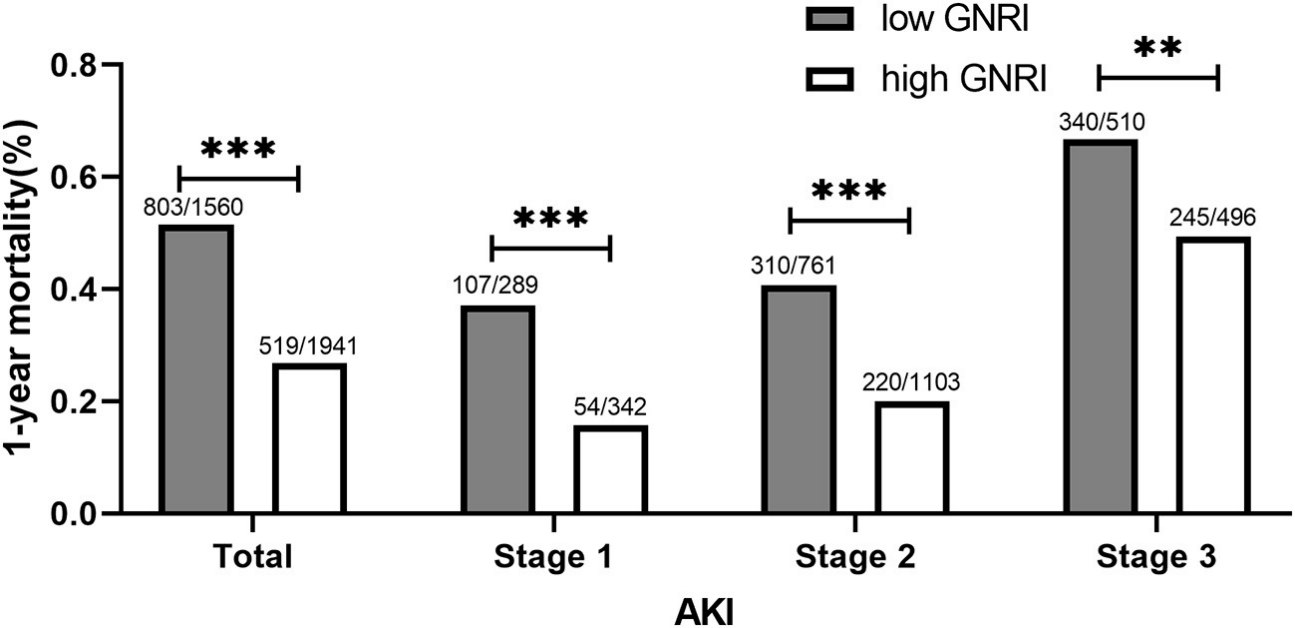
1-year mortality at different AKI stages between high and low GNRI groups. GNRI, geriatric nutritional risk index; AKI, acute kidney injury. ^**^P < 0.01; ^***^P < 0.001.

**Figure 2:**
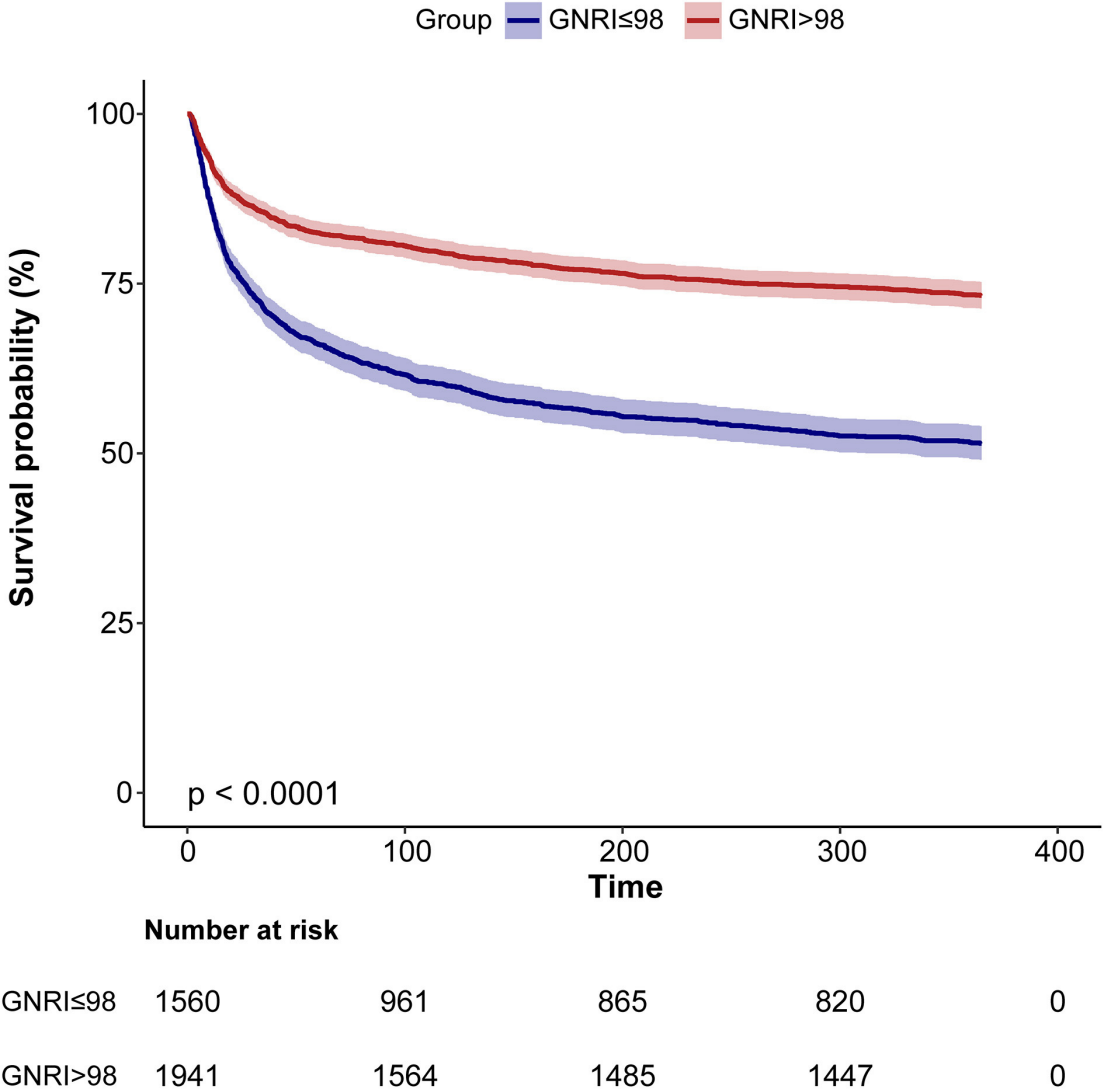
Kaplan–Meier curves for 1-year accumulative survival rates grouped by high and low GNRI. GNRI, geriatric nutritional risk index.

When stratified by the AKI stage, patients with high GNRI at AKI stages 1, 2, and 3 had markedly lower 1-year mortality than those with low GNRI (all *p* < 0.05, Figure 1). The KM curves for 365-day cumulative survival are presented in Figure 2, showing the significant survival advantage for patients with increased GNRI compared with those with decreased GNRI (log-rank *p* < 0.001).

**Figure 3:**
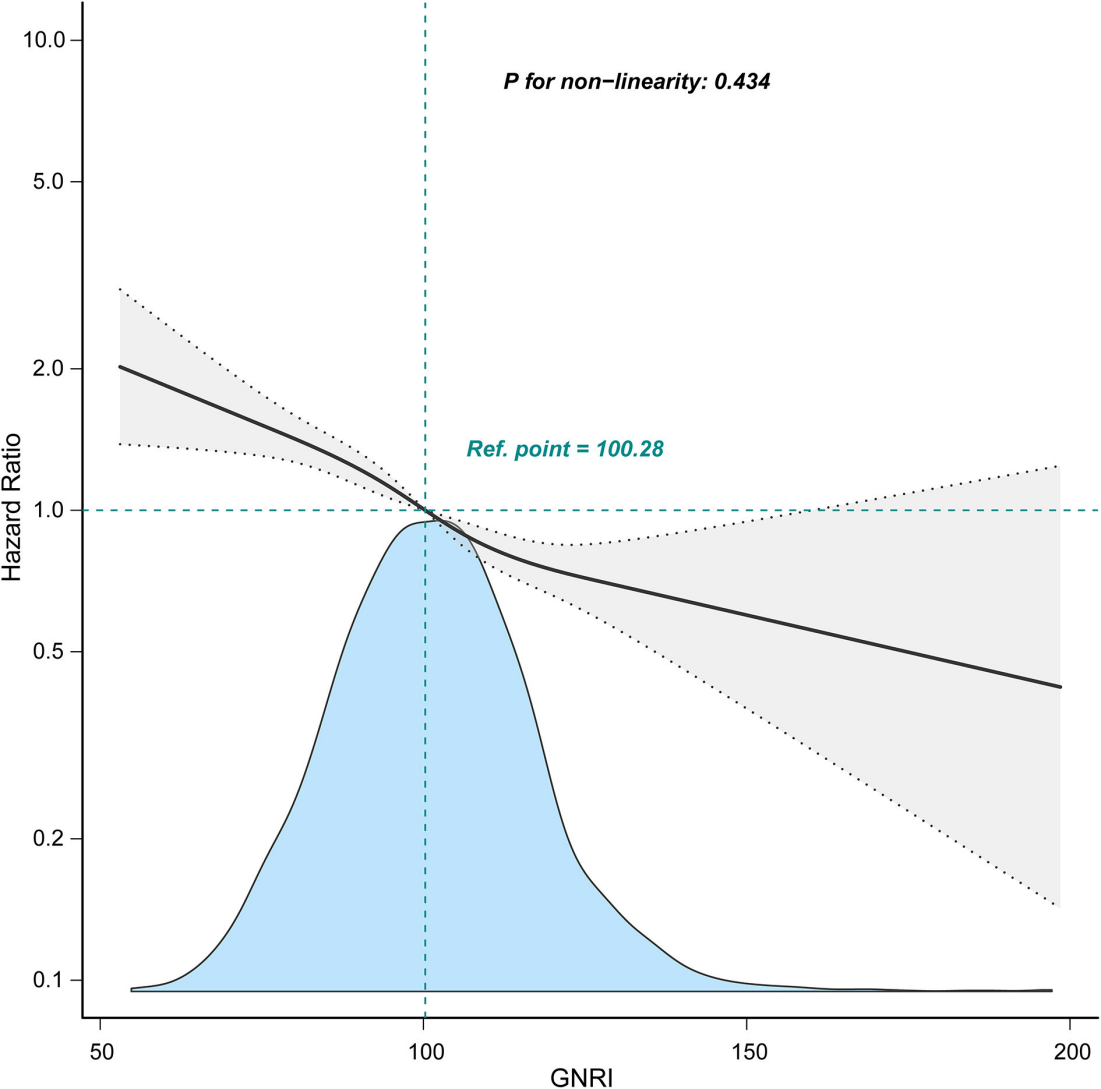
Relationship between GNRI and 1-year mortality using restricted cubic spline analysis. A linear relationship between GNRI and 1-year mortality was observed after adjusting for age, gender, heart rate, hypertension, diabetes, CHD, heart failure, CKD, liver cirrhosis, malignancy, sepsis, WBC, hemoglobin, BUN, SCr, sodium, potassium, anion gap, bicarbonate, ALT, AST, SOFA score, CRRT, and ventilation. GNRI, geriatric nutritional risk index.

Next, the multivariable regression analysis was conducted to confirm the independent relationship between GNRI and the worse outcomes. A one-unit increment of GNRI was remarkably associated with a lower risk of clinical outcomes after potential confounding factor adjustment (all *p* ≤ 0.001, Table 3), and these covariates (including age, gender, systolic blood pressure, heart rate, hypertension, diabetes, CHD, heart failure, liver cirrhosis, malignancy, sepsis, WBC, hemoglobin, BUN, SCr, sodium, potassium, anion gap, bicarbonate, alanine aminotransferase, AST, SOFA score, CCI, ICU types, CRRT, and ventilation) were significant in the univariable Cox regression analyses (Supplementary Table 1). Patients with elevated GNRI also had markedly reduced risk of adverse outcomes even after the potential confounding factors were controlled [OR (95% CI): 0.63 (0.51–0.77) for in-hospital mortality; OR (95% CI): 0.70 (0.56–0.87) for ICU mortality; OR (95% CI): 0.60 (0.50–0.73) for 28-day death; OR (95% CI): 0.59 (0.49–0.71) for 90-day death; OR (95% CI): 0.66 (0.54–0.79) for hospital stay ≥ 19 days; OR (95% CI): 0.62 (0.51–0.76) for ICU stay ≥ 10 days; and HR (95% CI): 0.68 (0.60–0.77) for 1-year mortality, Table 3]. RCS verified a linear association between GNRI and 1-year mortality (p for non-linearity = 0.434, Figure 3).

**Table 3:** Adjusted OR/HR of GNRI for adverse outcomes.

	**Univariable analysis**		**Multivariable analysis**	
	**OR/HR (95%Cl)**	***P***	**OR/HR(95%Cl)**	***P***
**GNRI as continuous variable**				
In-hospital death	0.97 (0.96–0.97)	<0.001	0.98 (0.97–0.99)	<0.001
ICU death	0.97 (0.97–0.98)	<0.001	0.99 (0.98–0.99)	<0.001
28-days death	0.97 (0.96–0.97)	<0.001	0.98 (0.97–0.99)	<0.001
90-days death	0.97 (0.96–0.97)	<0.001	0.98 (0.97–0.99)	<0.001
Hospital stay ≥19 days	0.98 (0.97–0.98)	<0.001	0.98 (0.98–0.99)	<0.001
ICU stay ≥10 days	0.98 (0.97–0.98)	<0.001	0.98 (0.98–0.99)	<0.001
1-year death	0.97 (0.97–0.98)	<0.001	0.98 (0.98–0.99)	<0.001
**GNRI as categorical variable**				
In-hospital death	0.41 (0.34–0.49)	<0.001	0.63 (0.51–0.77)	<0.001
ICU death	0.46 (0.39–0.56)	<0.001	0.70 (0.56–0.87)	0.002
28-days death	0.41 (0.35–0.48)	<0.001	0.60 (0.50–0.73)	<0.001
90-days death	0.39 (0.33–0.45)	<0.001	0.59 (0.49–0.71)	<0.001
Hospital stay ≥19 days	0.51 (0.44–0.60)	<0.001	0.66 (0.54–0.79)	<0.001
ICU stay ≥10 days	0.49 (0.42–0.58)	<0.001	0.62 (0.51–0.76)	<0.001
1-year death	0.47 (0.42–0.52)	<0.001	0.68 (0.60–0.77)	<0.001

Multivariable analysis adjusted for age, gender, systolic blood pressure, heart rate, hypertension, diabetes, CHD, heart failure, liver cirrhosis, malignancy, sepsis, WBC, hemoglobin, BUN, SCr, sodium, potassium, anion gap, bicarbonate, alanine aminotransferase, AST, SOFA score, CCI, ICU types, CRRT, and ventilation.

OR, odds ratio; HR, hazard ratio; other abbreviations as in Table 1.

### 3.3. Subgroup analyses

**Figure 4:**
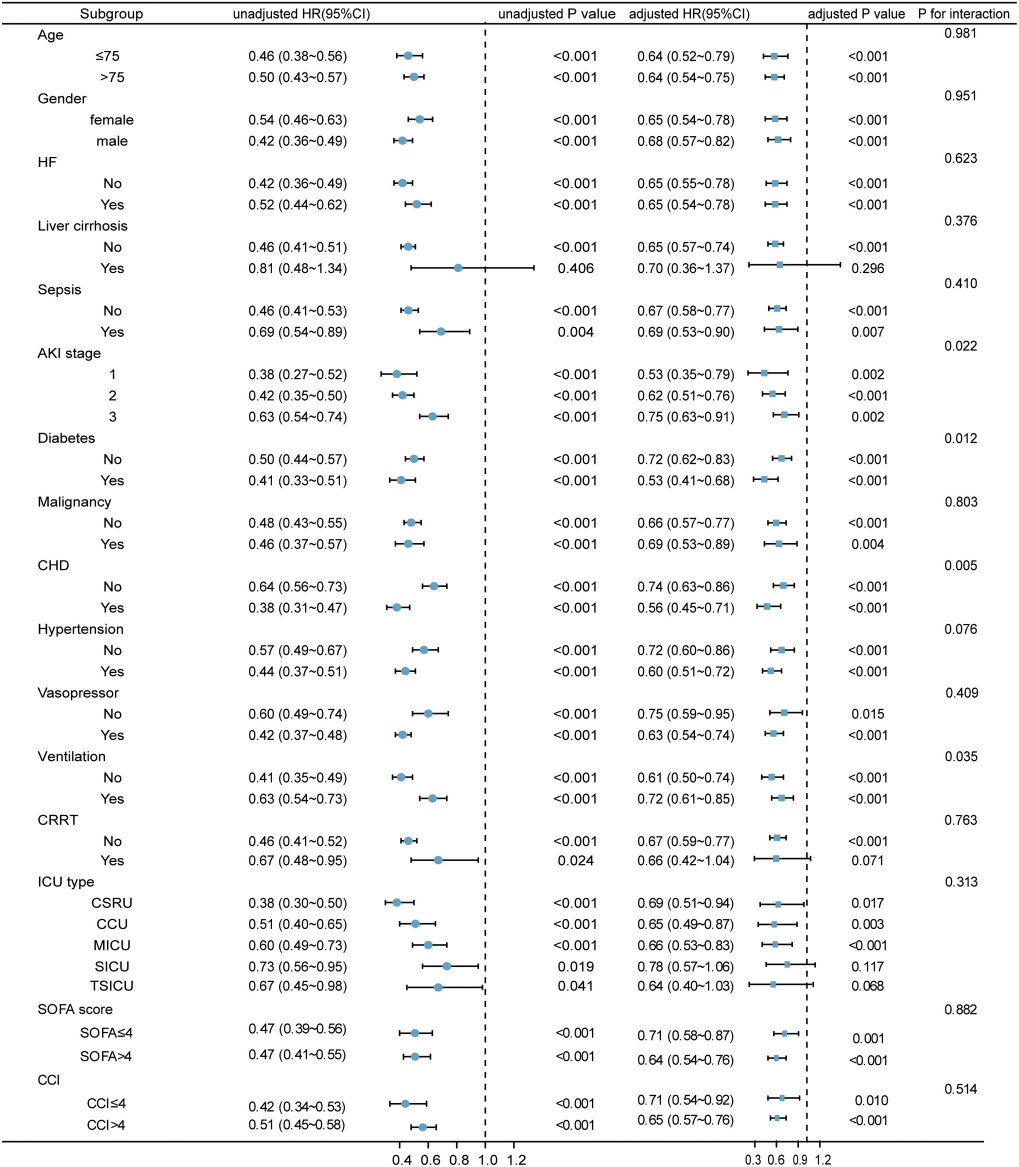
Relationship between GNRI and 1-year mortality in various subgroups. The HR was examined regarding the low GNRI as reference. HF, heart failure; AKI, acute kidney injury; CKD, chronic kidney disease; CHD, coronary heart disease; CRRT, continuous renal replacement therapy; GNRI, geriatric nutritional risk index; HR, hazard ratio; 95% CI, 95% confidence interval.

We further carried out subgroup analyses to figure out whether the correlation between GNRI and 1-year death was constant in various subclasses (Figure 4). GNRI still independently predicted 1-year death in patients with the most subgroups. Notably, the predictive implication of GNRI appeared to be more pronounced in patients with AKI stages 1 and 2 (P_interaction_ = 0.022), diabetes (P_interaction_ = 0.012), and CHD (P_interaction_ = 0.005) and also patients who did not receive mechanical ventilation (P_interaction_ = 0.035).

## 4. Discussion

Our study confirmed a negative linear correlation between GNRI and 1-year death in elderly AKI patients in the ICU, and upon an increase in GNRI, the mortality rate gradually decreased. GNRI at admission was remarkably related to a lower risk of research outcomes even after the covariates were controlled. Moreover, the relationship between GNRI and long-term mortality remained significant irrespective of the AKI stages.

At present, the common nutritional screening tools recommended for the older population include the Mini-Nutritional Assessment (MNA), Subjective Global Assessment (SGA), and Nutritional Risk Screening 2002 (NRS-2002) (20). Research studies have verified that SGA and NRS-2002 were vital markers for predicting poor prognosis in AKI patients (21, 22). No studies were found on MNA and AKI prognosis. These nutritional scoring tools are easily influenced by a subjective evaluation by trained professionals, particularly for questions regarding weight loss and dietary intake changes. These questions are too complex and cumbersome for critically ill older patients, which results in an inaccurate assessment of the patient's nutritional status. GNRI is a straightforward, objective, and well-established nutritional screening tool specially developed for the hospitalized elderly (18). Our analysis exhibited that decreased GNRI was remarkably associated with elevated mortality risk and prolonged stay length. This was in agreement with the results of the previously published studies: Malnourished AKI patients revealed lower survival rates (21–23). The BMI and ALB, components of GNRI, demonstrated a strong correlation with the mortality of critically ill AKI patients (24, 25). The present study extends the population to the elderly. More importantly, GNRI was consistently significantly related to long-term mortality irrespective of the AKI stage. Therefore, we recommend using GNRI to accurately assess the nutritional status of elderly patients with severe AKI, to identify high-risk malnutrition patients and provide nutritional guidance.

It has been well-established that comorbidities, such as sepsis, malignancy, heart failure, and liver cirrhosis, can further aggravate the nutritional status of patients in multiple ways (26). Nutritional status becomes worse with advancing age (27). Interventions, including CRRT, vasopressors, and mechanical ventilation, have been independently related to the mortality of severe AKI patients (28). Our results exhibited that the prognostic implication of GNRI remained significant in most of the subgroups, which indicated that the correlation between GNRI and long-term mortality could be generalized to different clinical settings. However, we could not explain why the predictive implication of GNRI was not significant in patients with CKD.

A higher GNRI indicates a lower nutritional risk. There was a negative linear relationship between GNRI and the research outcomes in the present analysis. High GNRI is caused by elevated albumin and BMI. The main pathological mechanisms of malnutrition in AKI patients are the reduced intake of nutrients and the loss of protein and energy related to metabolic disorders (4, 29), which lead to a decrease in BMI and albumin. Patients with high BMI tend to have better outcomes. This theory is called the obesity paradox, which has been proved in patients with AKI (25), CHD (30, 31), and heart failure (32) and in those receiving hemodialysis (33). Another mechanism that explains the association between albumin and malnutrition in patients with kidney disease is that albumin possesses anti-inflammatory and antioxidant properties (34, 35). It has been documented that inflammatory cascades and oxidative stress play an essential role in the progression of malnutrition (29, 36).

Our analysis still existed certain limitations. Although the data in this study were obtained from large public databases, no external verification was conducted to verify the predictive significance of GNRI. Second, GNRI was assessed only at admission. Further research should be conducted to explore the prognostic implication of the dynamic change of GNRI. Third, since MIMIC III is based on data from 2001 to 2012, our research may not be fully generalizable to current medical conditions. Next, because of the limitations of the MIMIC database, we could not collect information on diet and physical activity, which was correlated with the nutritional status of patients. Finally, we cannot clarify the reason for AKI due to the limitations of the MIMIC database.

## 5. Conclusion

On-admission GNRI is a pivotal predictor of the adverse endpoints of elderly AKI patients in the ICU. Our results suggested that GNRI helped to identify elderly AKI patients at a high risk of malnutrition to ensure timely and effective nutritional support.

## Data availability statement

Publicly available datasets were analyzed in this study. This data can be found here: https://mimic-iv.mit.edu.

## Author contributions

DL was responsible for designing the protocol, conducting the search and analyzing data from MIMIC-III, interpreting results, and creating summary of findings tables. YD, XL, JH, and JL were responsible for designing the review protocol and extracting data. YD, XL, and MP contributed to updating reference lists and provided feedback on the report. FZ and LW contributed to analyzing data, interpreting results, as well as creating tables and figures, and writing the paper. All authors contributed to the article and approved the submitted version.

## References

[1] Hoste EA Bagshaw SM Bellomo R Cely CM Colman R Cruz DN. Epidemiology of acute kidney injury in critically ill patients: the multinational AKI-EPI study. Intensive Care Med. (2015) 41:1411–23. 10.1007/s00134-015-3934-7 26162677

[2] Meyer D Mohan A Subev E Sarav M Sturgill D. Acute kidney injury incidence in hospitalized patients and implications for nutrition support. Nutr Clin Pract. (2020) 35:987–1000. 10.1002/ncp.10595 33140897

[3] Bellomo R Cass A Cole L Finfer S Gallagher M Lee J. Calorie intake and patient outcomes in severe acute kidney injury: findings from the randomized evaluation of normal vs. augmented level of replacement therapy (RENAL) study trial. Crit Care. (2014) 18:R45. 10.1186/cc13767 24629036 PMC4057152

[4] Fiaccadori E Sabatino A Barazzoni R Carrero JJ Cupisti A De Waele E. ESPEN guideline on clinical nutrition in hospitalized patients with acute or chronic kidney disease. Clin Nutr. (2021) 40:1644–68. 10.1016/j.clnu.2021.01.028 33640205

[5] Fiaccadori E Regolisti G Maggiore U. Specialized nutritional support interventions in critically ill patients on renal replacement therapy. Curr Opin Clin Nutr Metab Care. (2013) 16:217–24. 10.1097/MCO.0b013e32835c20b0 23242314

[6] Guenter P Abdelhadi R Anthony P Blackmer A Malone A Mirtallo JM. Malnutrition diagnoses and associated outcomes in hospitalized patients: United States, 2018. Nutr Clin Pract. (2021) 36:957–69. 10.1002/ncp.10771 34486169

[7] Barker LA Gout BS Crowe TC. Hospital malnutrition: prevalence, identification and impact on patients and the healthcare system. Int J Environ Res Public Health. (2011) 8:514–27. 10.3390/ijerph8020514 21556200 PMC3084475

[8] Agarwal E Ferguson M Banks M Bauer J Capra S Isenring E. Nutritional status and dietary intake of acute care patients: results from the Nutrition Care Day Survey 2010. Clin Nutr. (2012) 31:41–7. 10.1016/j.clnu.2011.08.002 21862187

[9] Xie H Tang S Wei L Gan J. Geriatric nutritional risk index as a predictor of complications and long-term outcomes in patients with gastrointestinal malignancy: a systematic review and meta-analysis. Cancer Cell Int. (2020) 20:530. 10.1186/s12935-020-01628-7 33292289 PMC7603782

[10] Ruan GT Zhang Q Zhang X Tang M Song MM Zhang XW. Geriatric Nutrition Risk Index: Prognostic factor related to inflammation in elderly patients with cancer cachexia. J Cachexia Sarcopenia Muscle. (2021) 12:1969–82. 10.1002/jcsm.12800 34585849 PMC8718015

[11] Nishi I Seo Y Hamada-Harimura Y Yamamoto M Ishizu T Sugano A. Geriatric nutritional risk index predicts all-cause deaths in heart failure with preserved ejection fraction. ESC Heart Fail. (2019) 6:396–405. 10.1002/ehf2.12405 30706996 PMC6437432

[12] Fan Y He L Zhou Y Man C. Predictive value of geriatric nutritional risk index in patients with coronary artery disease: a meta-analysis. Front Nutr. (2021) 8:736884. 10.3389/fnut.2021.736884 34660665 PMC8511313

[13] Yuan K Zhu S Wang H Chen J Zhang X Xu P. Association between malnutrition and long-term mortality in older adults with ischemic stroke. Clin Nutr. (2021) 40:2535–42. 10.1016/j.clnu.2021.04.018 33932800

[14] Shi X Shen Y Yang J Du W Yang J. The relationship of the geriatric nutritional risk index to mortality and length of stay in elderly patients with acute respiratory failure: a retrospective cohort study. Heart Lung. (2021) 50:898–905. 10.1016/j.hrtlng.2021.07.012 34411871

[15] Shao Y Lai QC Duan Q Ge P Ye L. Nutritional indices at admission are associated with mortality rates of patients in the intensive care unit. Eur J Clin Nutr. (2022) 76:557–63. 10.1038/s41430-021-00994-3 34404932

[16] Nakagawa N Maruyama K Hasebe N. Utility of geriatric nutritional risk index in patients with chronic kidney disease: a mini-review. Nutrients. (2021) 13:3688. 10.3390/nu13113688 34835944 PMC8624060

[17] Panichi V Cupisti A Rosati A Di Giorgio A Scatena A Menconi O. Geriatric nutritional risk index is a strong predictor of mortality in hemodialysis patients: data from the Riscavid cohort. J Nephrol. (2014) 27:193–201. 10.1007/s40620-013-0033-0 24430765

[18] Bouillanne O Morineau G Dupont C Coulombel I Vincent JP Nicolis I. Geriatric Nutritional Risk Index: a new index for evaluating at-risk elderly medical patients. Am J Clin Nutr. (2005) 82:777–83. 10.1093/ajcn/82.4.777 16210706

[19] Ad-hoc Ad-hoc working group of E Fliser D Laville M Covic A Fouque D Vanholder R. A European renal best practice (ERBP) position statement on the Kidney Disease Improving Global Outcomes (KDIGO) clinical practice guidelines on acute kidney injury: part 1: definitions, conservative management and contrast-induced nephropathy. Nephrol Dial Transplant. (2012) 27:4263–72. 10.1093/ndt/gfs375 23045432 PMC3520085

[20] Abd Aziz NAS Teng N Abdul Hamid MR Ismail NH. Assessing the nutritional status of hospitalized elderly. Clin Interv Aging. (2017) 12:1615–25. 10.2147/CIA.S140859 29042762 PMC5634387

[21] Khor BH Tiong HC Tan SC Abdul Rahman R Abdul Gafor AH. Protein-energy wasting assessment and clinical outcomes in patients with acute kidney injury: a systematic review with meta-analysis. Nutrients. (2020) 12:2809. 10.3390/nu12092809 32933198 PMC7551057

[22] Li C Xu L Guan C Zhao L Luo C Zhou B. Malnutrition screening and acute kidney injury in hospitalised patients: a retrospective study over a 5-year period from China. Br J Nutr. (2020) 123:337–46. 10.1017/S000711451900271X 31657292

[23] Wang N Wang P Li W Jiang L Wang M Zhu B. Prognostic significance of malnutrition risk in elderly patients with acute kidney injury in the intensive care unit. BMC Nephrol. (2022) 23:335. 10.1186/s12882-022-02949-7 36258183 PMC9578231

[24] Lv J Wang H Sun B Gao Y Zhang Z Pei H. Serum albumin Before CRRT was associated with the 28- and 90-day mortality of critically ill patients with acute kidney injury and treated with continuous renal replacement therapy. Front Nutr. (2021) 8:717918. 10.3389/fnut.2021.717918 34513902 PMC8425552

[25] Wang B Li D Gong Y Ying B Cheng B Sun L. Body mass index is associated with the severity and all-cause mortality of acute kidney injury in critically ill patients: an analysis of a large critical care database. Biomed Res Int. (2021) 2021:6616120. 10.1155/2021/6616120 34258271 PMC8260311

[26] Norman K Pichard C Lochs H Pirlich M. Prognostic impact of disease-related malnutrition. Clin Nutr. (2008) 27:5–15. 10.1016/j.clnu.2007.10.007 18061312

[27] Hickson M. Malnutrition and ageing. Postgrad Med J. (2006) 82:2–8. 10.1136/pgmj.2005.037564 16397072 PMC2563720

[28] Uchino S Kellum JA Bellomo R Doig GS Morimatsu H Morgera S. Acute renal failure in critically ill patients: a multinational, multicenter study. JAMA. (2005) 294:813–8. 10.1001/jama.294.7.813 16106006

[29] Sabatino A Regolisti G Karupaiah T Sahathevan S Sadu Singh BK Khor BH. Protein-energy wasting and nutritional supplementation in patients with end-stage renal disease on hemodialysis. Clin Nutr. (2017) 36:663–71. 10.1016/j.clnu.2016.06.007 27371993

[30] Lavie CJ Milani RV Artham SM Patel DA Ventura HO. The obesity paradox, weight loss, and coronary disease. Am J Med. (2009) 122:1106–14. 10.1016/j.amjmed.2009.06.006 19682667

[31] Niedziela J Hudzik B Niedziela N Gasior M Gierlotka M Wasilewski J. The obesity paradox in acute coronary syndrome: a meta-analysis. Eur J Epidemiol. (2014) 29:801–12. 10.1007/s10654-014-9961-9 25354991 PMC4220102

[32] Oreopoulos A Padwal R Kalantar-Zadeh K Fonarow GC Norris CM McAlister FA. Body mass index and mortality in heart failure: a meta-analysis. Am Heart J. (2008) 156:13–22. 10.1016/j.ahj.2008.02.014 18585492

[33] Kalantar-Zadeh K Streja E Kovesdy CP Oreopoulos A Noori N Jing J. The obesity paradox and mortality associated with surrogates of body size and muscle mass in patients receiving hemodialysis. Mayo Clin Proc. (2010) 85:991–1001. 10.4065/mcp.2010.0336 21037042 PMC2966362

[34] Roche M Rondeau P Singh NR Tarnus E Bourdon E. The antioxidant properties of serum albumin. FEBS Lett. (2008) 582:1783–7. 10.1016/j.febslet.2008.04.057 18474236

[35] Don BR Kaysen G. Serum albumin: relationship to inflammation and nutrition. Semin Dial. (2004) 17:432–7. 10.1111/j.0894-0959.2004.17603.x 15660573

[36] Saha SK Lee SB Won J Choi HY Kim K Yang GM. Correlation between oxidative stress, nutrition, and cancer initiation. Int J Mol Sci. (2017) 18:1544. 10.3390/ijms18071544 28714931 PMC5536032

