# Supplementary material for: The prognostic effects of the geriatric nutritional risk index on elderly acute kidney injury patients in intensive care units
Source: Front Med (Lausanne). 2023 May 11;10:1165428. doi: 10.3389/fmed.2023.1165428 (PMC10213743; doi:10.3389/fmed.2023.1165428)
Supplement: Supplementary file 1 [file Table_1.DOC]

**Supplementary table 1** Univariable regression analyses for 1-year mortality.

|  | HR (95%CI) | *P* |
| --- | --- | --- |
| Age, years | 1.03 (1.03, 1.04) | <0.001 |
| Male, n (%) | 0.89 (0.80, 0.99) | 0.039 |
| SBP, mmHg | 1.00 (1.00, 1.00) | 0.041 |
| DBP, mmHg | 1.01 (1.00, 1.01) | 0.005 |
| Heart rate, bpm | 1.01 (1.01, 1.02) | <0.001 |
| Hypertension, n (%) | 0.62 (0.56, 0.69) | <0.001 |
| Diabetes, n (%) | 0.80 (0.70, 0.91) | <0.001 |
| CHD, n (%) | 0.43 (0.38, 0.48) | <0.001 |
| Heart failure, n (%) | 1.27 (1.14, 1.42) | <0.001 |
| Liver cirrhosis, n (%) | 1.48 (1.14, 1.93) | 0.003 |
| Malignancy, n (%) | 1.47 (1.30, 1.66) | <0.001 |
| Sepsis,n (%) | 2.40 (2.10, 2.74) | <0.001 |
| WBC, k/ul | 1.04 (1.03, 1.04) | <0.001 |
| Hemoglobin, g/dl | 0.92 (0.90, 0.95) | <0.001 |
| BUN, mg/dl | 1.01 (1.01, 1.01) | <0.001 |
| SCr, mg/dl | 1.21 (1.16, 1.27) | <0.001 |
| Sodium, mEq/l | 0.97 (0.96, 0.99) | <0.001 |
| Potassium, mEq/l | 1.29 (1.21, 1.37) | <0.001 |
| Anion gap,mEq/L | 1.08 (1.06, 1.09) | <0.001 |
| Bicarbonate, mEq/L | 0.97 (0.96, 0.98) | <0.001 |
| ALT, u/l | 1.00 (1.00, 1.00) | <0.001 |
| AST, u/l | 1.00 (1.00, 1.00) | <0.001 |
| GNRI as continuous variable | 0.97 (0.97, 0.98) | <0.001 |
| GNRI as categorical variable | 0.47 (0.42, 0.52) | <0.001 |
| SOFA score | 1.07 (1.05, 1.09) | <0.001 |
| CRRT, n (%) | 2.47 (2.06, 2.96) | <0.001 |
| CCI | 1.22 (1.19, 1.25) | < 0.001 |
| ICU types |  |  |
| CSRU | Reference | Reference |
| CCU | 2.99 (2.51, 3.58) | <0.001 |
| MICU | 3.93 (3.35, 4.62) | <0.001 |
| SICU | 3.20 (2.66, 3.85) | <0.001 |
| TSICU | 2.79 (2.22, 3.51) | <0.001 |
| Vasopressors, n (%) | 1.06 (0.94, 1.20) | 0.340 |
| Ventilation, n (%) | 2.52 (2.25, 2.81) | <0.001 |

GNRI, geriatric nutritional risk index; SBP, systolic blood pressure; DBP, diastolic blood pressure; CHD, coronary heart disease; WBC, white blood cell count; BUN, blood urea nitrogen; SCr, serum creatinine; ALT, alanine aminotransferase; AST, aspartate aminotransferase; SOFA, sequential organ failure assessment; CCI, charlson comorbidity index; CRRT, continuous renal replacement therapy; ICU, intensive care unit; CCU, coronary care unit; CSRU, cardiothoracic surgery recovery unit; MICU, medical intensive care unit; SICU, surgical intensive care unit; TSICU, trauma and surgical intensive care unit.
