# Supplementary material for: The prognostic effects of the geriatric nutritional risk index on elderly acute kidney injury patients in intensive care units
Source: Front Med (Lausanne). 2023 May 11;10:1165428. doi: 10.3389/fmed.2023.1165428 (PMC10213743; doi:10.3389/fmed.2023.1165428)
Supplement: Supplementary file 2 [file Table_2.DOCX]

**
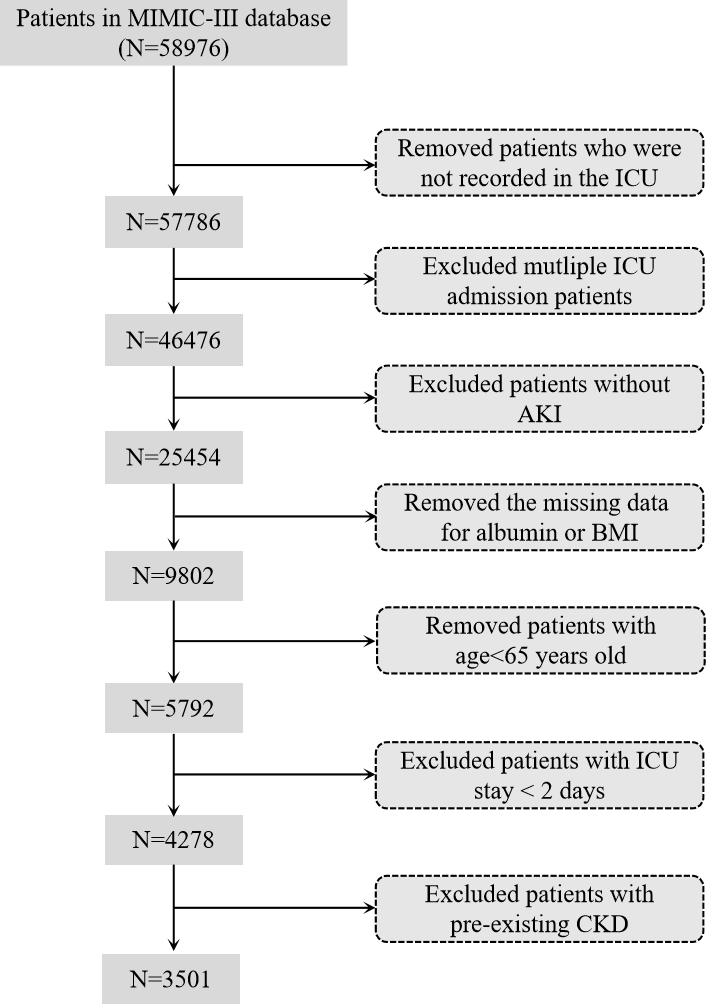
**

**Supplementary Figure 1.** Flow chart of the study population enrollment**.** MIMIC, Medical Information Mart for Intensive Care; ICU, intensive care unit; AKI, acute kidney injury; BMI, Body Mass Index; CKD, chronic kidney disease.

**
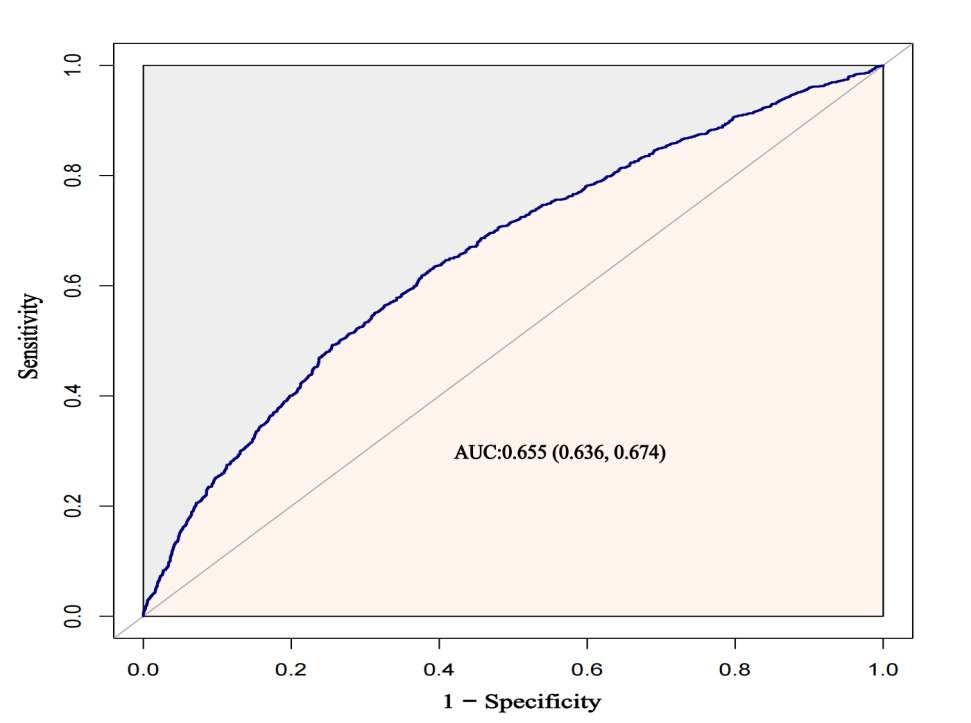
**

**Supplementary Figure 2.** The receiver operating characteristic curve of GNRI for predicting 1-year mortality in MIMIC III. The best cut-off value was ≤ 98. The area under the curve was 0.655, and the sensitivity and specificity were 0.618 and 0.623, respectively.
